# Review of Subcutaneous Wound Drainage in Reducing Surgical Site Infections after Laparotomy

**DOI:** 10.1155/2015/715803

**Published:** 2015-12-13

**Authors:** B. Manzoor, N. Heywood, A. Sharma

**Affiliations:** ^1^Department of Surgery, University Hospital of South Manchester and The University of Manchester, MAHSC, Manchester, UK; ^2^Wythenshawe Hospital, University Hospital of South Manchester, Southmoor Road, Manchester M23 9LT, UK

## Abstract

*Purpose.* Surgical site infections (SSIs) remain a significant problem after laparotomies. The aim of this review was to assess the evidence on the efficacy of subcutaneous wound drainage in reducing SSI.* Methods*. MEDLINE database was searched. Studies were identified and screened according to criteria to determine their eligibility for meta-analysis. Meta-analysis was performed using the Mantel-Haenszel method and a fixed effects model.* Results*. Eleven studies were included with two thousand eight hundred and sixty-four patients. One thousand four hundred and fifty patients were in the control group and one thousand four hundred and fourteen patients were in the drain group. Wound drainage in all patients shows no statistically significant benefit in reducing SSI incidence. Use of drainage in high risk patients, contaminated wound types, and obese patients appears beneficial.* Conclusion*. Using subcutaneous wound drainage after laparotomy in all patients is unnecessary as it does not reduce SSI risk. Similarly, there seems to be no benefit in using it in clean and clean contaminated wounds. However, there may be benefit in using drains in patients who are at high risk, including patients who are obese and/or have contaminated wound types. A well designed trial is needed which examines these factors.

## 1. Introduction

Surgical site infections (SSIs) are defined as wound infection following an invasive surgical procedure [1]. These remain a substantial problem for patients undergoing procedures in spite of advances in surgical techniques and medical care.

SSIs have been shown to contribute up to 20% of nosocomial infections with an overall incidence around 5% across all invasive surgical procedures [1]. Laparotomies carry a higher risk of wound infection and a combined rate of 15% has been reported in upper and lower gastrointestinal surgery, over three times the average risk [2]. Furthermore, in large bowel surgery, an overall infection rate of 17.5% has been identified in the UK [3, 4]. Rates as high as 26% in colorectal procedures [5] and up to 57% in small bowel procedures [6] have also been described.

SSIs lead to increased hospital stay and increased morbidity [7] alongside increasing unnecessary patient suffering and a decreased quality of life (QoL) [8, 9]. A recent study done in Japan identified an increase of mean hospital stay by 17.8 days in patients who developed SSI after colorectal surgery [10] and similarly a 13.2-day length of stay increase following small bowel surgery has also been described [11]. When combining these with the costs of treating the SSIs, in the UK they have been shown to account for up to an extra £700 million of the NHS health budget annually [12, 13].

Numerous risk factors for developing a SSI have been identified. Current smokers are at a 30% increased risk of SSI after major colorectal procedures [14] and smoking cessation reduces SSI [15]. Body Mass Index and obesity have also been linked to increased risk of SSI [16] with studies showing wound complication rates in some procedures rising from 7% up to 23% due to obesity [17]. More specifically, depth of subcutaneous fat has been shown to be a strong risk factor for SSI [18] and has been shown to be a useful predictor for SSI risk [19]. Many other factors including nutrition and diabetes control, certain comorbidities, ASA class, and operation time have been identified as important factors affecting SSI [19, 20].

Various interventions have been proposed with a view to reducing SSIs. A number of them are used in routine practice. Hand washing, minimising shaving, skin preparation, and preoperative antibiotics have all gained acceptance in the surgical community [21–24]. Use of drains after surgery however has declined in recent times. It has been shown that drains provide no advantage after cholecystectomies, inguinal hernia repairs, and various other types of surgery [25]. Use of drains, however, is still popular after abdominoperineal excision of rectum and repair of incisional hernias due to inconclusive evidence and surgeon preference [26, 27]. They are still used in some major plastic surgery procedures as they are thought to reduce collections in closed spaces [28].

It has been postulated that the presence of haematoma, serous fluid, and dead space in surgical incisional wounds increases the risk of infection as this acts as a culture medium [29, 30]. Subcutaneous drains have been used to reduce the risk of infection [31]. However, the use of postoperative subcutaneous wound drainage is not universally accepted. In addition drains may not be efficacious and cause discomfort and increased hospital stay on their own [32].

The aim of this systematic review is to assimilate and analyse the available evidence regarding the efficacy of subcutaneous wound drainage in reducing s-SSI after laparotomy.

## 2. Method

A search of the MEDLINE database through PubMed was performed with the aim of identifying articles regarding the primary search criteria,* Superficial abdominal wound drainage and the impact on wound infection.* Articles were considered from any country and any year but articles that did not meet the language criteria (English) were going to be excluded; however no articles were found that did not meet the language criteria at the end of our screening process.

Search was performed using the terms “*subcutaneous wound drainage*” and “*drain AND subcutaneous AND infection*”. All the abstracts were considered against the primary search criteria and 48 articles were retrieved. The articles were then screened for duplicates and 19 articles were highlighted and were removed, leaving a total of 29 articles.

An additional 2 articles were retrieved after reviewing references from these bringing the total number of articles after primary screening to 31.

**Figure 1:**
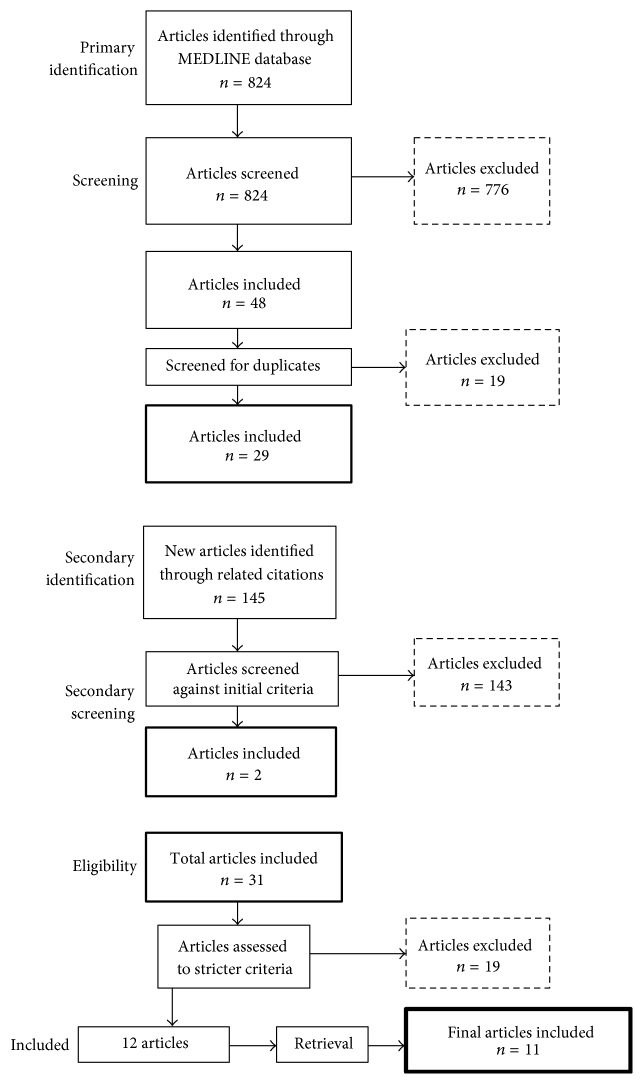
Flow chart showing the method of identifying eligible articles for the purpose of our analysis.

The retrieved articles were then put through the secondary screening. The articles were screened against the criteria “*primary incision must be a true laparotomy*”. Gynaecological procedures and Caesarean sections alongside other nonlaparotomy abdominal incisions were excluded. A total of 19 articles were excluded which left 12 articles. One of the 12 articles was a meta-analysis [25] leaving* 11 articles* for the review (Figure 1).

The relevant data for the purpose of this systematic review was extracted from each trial. Chi-squared analysis of each individual trial was performed to determine significance. The data was then used to perform a meta-analysis. The Mantel-Haenszel method was used with a fixed effects model to determine risk ratios (RR) and confidence intervals (CI) for each individual trial in addition to an overall RR, CI, and *P* value for the collated data.

## 3. Results

**Figure 2:**
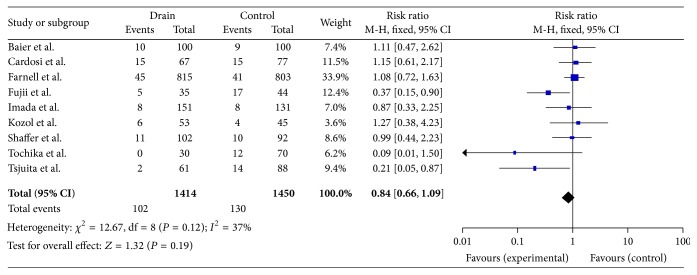
Forest plot data comparing ten trials from Table 1.

Two thousand eight hundred and sixty-four patients undergoing laparotomies in nine different trials were included in this meta-analysis [32–42] (Table 1). Two studies (Table 2) included some nonlaparotomy incisions and were analysed separately. On meta-analysis (Figure 2), the trials were found to be homogenous (*P* value of 0.12); therefore the data from the trials was collated and analysed using a fixed effects model.

Chi-squared analysis was used on each of the trials using 95% confidence intervals. Three trials showed a significant reduction in surgical site infections in the drainage group. On assessing the risk ratios and respective confidence intervals, only two showed a significant reduction in SSI in the drain group as opposed to the control group.

Overall no significant difference was found in the SSI rate in the two groups (*P* = 0.19, risk ratio 0.84 (0.66–1.09)).

**Figure 3:**
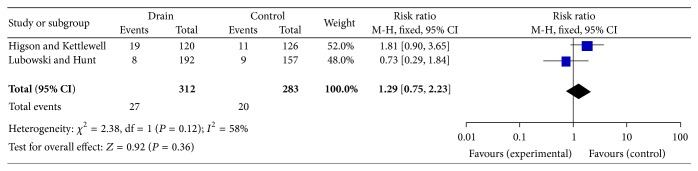
Forest plot data for trials including nonlaparotomy incisions.

Two studies with some nonlaparotomy incision were analysed separately. Higson et al. showed a significantly higher infection rate in the drain group compared to the control group. However, on meta-analysis (Figure 3), an overall *P* value of 0.36 was found [RR 1.29 (0.75–2.23)].

Table 3 shows data extracted from Farnell et al. and Lubowski et al. in which wound type was classified in both the control and drain groups. There was a reduction in both trials of the rate of wound infection in the contaminated wound type when using a drain as opposed to a control group; however the overall risk ratio was not significant (RR 0.56 (0.21–1.51)).

## 4. Discussion

The aim of this study was to do a systematic review of the evidence available on the use of subcutaneous wound drainage after laparotomies to determine if there is a reduction in the incidence of surgical site infections (SSIs).

We only included studies with laparotomy incisions in this review. The aim was to include a homogenous group of studies which could be compared and data combined to perform a meta-analysis. Incidence of SSI is higher in laparotomies compared to hernia operations or pfannenstiel incisions and this is accentuated further in emergency laparotomies. A recent systematic review and meta-analyses by Kosin et al. looking at subcutaneous wound drainage for a variety of incisions showed that drains could be omitted in most procedures but there was no specific focus on laparotomies. Two trials analysed the types of surgery (clean, clean contaminated, contaminated, and dirty) separately (Table 3). There was no statistically significant difference in the groups in these trials.

There was no significant reduction in SSI incidence when all the laparotomies were analysed together in our meta-analyses. The risk ratio determined was 0.84 (0.66–1.09) which cannot be taken as a reliable indication about the efficacy of using drains. Two trials were analysed separately. Higson et al. and Lubowski et al. trials showed no significant difference either in the rate of SSI. Higson et al. showed an almost double infection rate in the drain group as opposed to the control group. However this difference was not statistically significant (*P* = 0.089). Alongside this, both trials consisted of a relatively small sample size; hence reliable conclusions cannot be formed on this alone.

Out of all the trials in the meta-analysis, only two trials showed a significant reduction in SSI incidence in the drain group. Fujii et al. included high risk patients, including emergency laparotomies, and patients with thick subcutaneous fat and the risk ratio showed a reduction in the SSI rate in the drain group (RR 0.37 (0.15–0.9)). Imada et al. showed no significant difference in SSI incidence when using a drain in all patients; however there was a reduction in SSIs in the high risk patient group from 15% to 8%. It has also been reported by Soper et al. [18] that the depth of subcutaneous fat in a patient is an independent risk factor for SSI. It may therefore be possible that subcutaneous drains may be of benefit in high risk and/or obese patients and this is not evident in the meta-analysis due to underpowering. Indeed two trials detailed the wound types in each of the control and the drain groups and in these trials there was an overall reduction of 44% in SSI in the contaminated wound type where a subcutaneous drain was placed.

Various newer potential interventions may be used to reduce SSIs in this group of patients. The recently concluded ROSSINI trial assesses the efficacy of using wound edge protection devices in reducing SSI rates in laparotomies [43]. The trial results have recently been presented (ACPGBI Liverpool 1st–3rd July 2013) and do not show any advantage of using wound protectors. The authors are currently designing a further trial to address some of the shortcomings of the trial. Wound wicks which are removed at 72 hours can be used to prevent subcutaneous collection and may be useful. The National Emergency Laparotomy Audit (NELA) is a new initiative in the United Kingdom to audit and subsequently reduce complication rates after emergency laparotomies [44]. SSIs remain a major problem after emergency laparotomies and would be within the remit of NELA. This would further highlight the significance of interventions that reduce SSI in emergency laparotomies.

We aimed at keeping the studies as homogenous as possible for a reliable systematic review and analysis but despite this, there are still many variables between the trials which may have had an influence on the results. Wound drainage in all patients does not seem to be of significant benefit in reducing SSI and may add up to unnecessary cost, discomfort, and prolonged postoperative stay.

However, there may be potential benefit in higher risk patients, patients with deeper subcutaneous fat, and patients with contaminated or dirty wounds. These individual factors need to be carefully investigated in patients who undergo laparotomy. Other novel devices are also available which utilise suction to reduce the formation of collections under wounds. These have not been evaluated in a controlled trial. Wound wicks may also be used to ensure drainage of wounds in the immediate postoperative period. There is a need for a randomised controlled trial with well defined inclusion/exclusion criteria to evaluate use of such interventions in patients undergoing emergency laparotomies.

## Figures and Tables

**Table 1:** Studies detailing the effects of subcutaneous wound drainage in laparotomies and detailing the outcome by infection rates.

Author	Year	Patients	Drain type	Control				Drain				Calculated *P* value
				Total	SSI	No SSI	% infec.	Total	SSI	No SSI	% infec.	CI 95%
Shaffer et al. [38]	1987	194	Closed suction	92	10	82	**10.9**	102	11	91	**10.8**	0.985
Fujii et al. [35]	2011	79	Open	44	17	27	**38.6**	35	5	30	**14.3**	0.017
Imada et al. [36]	2013	282	Open	131	8	123	**6.1**	151	8	143	**5.3**	0.770
Tochika et al. [39]	2011	100	Closed suction	70	12	58	**17.1**	30	0	30	**0.0**	0.016
Cardosi et al. [33]	2006	144	Closed suction	77	15	62	**17.5**	67	15	52	**22.4**	0.668
Baier et al. [32]	2010	200	Closed suction	100	9	91	**9.0**	100	10	90	**10.0**	0.809
Tsujita et al. [40]	2012	149	Open	88	14	74	**15.9**	61	2	59	**3.3**	0.014
Kozol et al. [37]	1986	98	Suction	45	4	41	**8.9**	53	6	47	**11.3**	0.692
Farnell et al. [34]	1986	1618	Suction	803	41	762	**5.1**	815	45	770	**5.5**	0.709

**Table 2:** Studies in which only the laparotomy data could not be extracted. Nonlaparotomy abdominal incisions were included.

Author	Year	Patients	Drain type	No drain				Drain				Chi-squared 1 DF 2-tailed
				Total	SSI	No SSI	% infec.	Total	SSI	No SSI	% infec.	*P* Value CI 0.05
Higson and Kettlewell [41]	1978	246	Open	126	11	115	8.7	120	19	101	15.8	0.089
Lubowski and Hunt [42]	1987	349	Closed suction	157	9	148	5.7	192	8	184	4.2	0.499

**Table 3:** Studies detailing wound type in the control and drain groups.

Author	Year	Control					Drain				
		Number of (%) infections					Number of (%) infections				
		Total patients	Clean	Clean contam.	Contaminated	Dirty	Total patients	Clean	Clean contam.	Contaminated	Dirty
Lubowski and Hunt [42]	1987	157	2 (2.8)	4 (5.1)	2 (33.3)	1 (33.3)	192	2 (2.6)	4 (3.8)	2 (20)	0 (0)
Farnell et al. [34]	1986	803	—	27 (4.1)	7 (7.1)	7 (15.1)	815	—	29 (4.4)	4 (3.9)	12 (22.6)
